# Immunocontraceptive target repertoire defined by systematic identification of sperm membrane alloantigens in a single species

**DOI:** 10.1371/journal.pone.0190891

**Published:** 2018-01-17

**Authors:** Nathaly Cormier, John J. McGlone, John Leszyk, Daniel M. Hardy

**Affiliations:** 1 Department of Cell Biology & Biochemistry, Texas Tech University Health Sciences Center, Lubbock, Texas, United States of America; 2 Department of Animal and Food Sciences, Texas Tech University, Lubbock, Texas, United States of America; 3 Proteomic and Mass Spectrometry Facility and Department of Biochemistry & Pharmacology, University of Massachusetts Medical School, Shrewsbury, Massachusetts, United States of America; Universite Clermont Auvergne, FRANCE

## Abstract

Sperm competence in animal fertilization requires the collective activities of numerous sperm-specific proteins that are typically alloimmunogenic in females. Consequently, sperm membrane alloantigens are potential targets for contraceptives that act by blocking the proteins’ functions in gamete interactions. Here we used a targeted proteomics approach to identify the major alloantigens in swine sperm membranes and lipid rafts, and thereby systematically defined the repertoire of these sperm-specific proteins in a single species. Gilts with high alloantibody reactivity to proteins in sperm membranes or lipid rafts produced fewer offspring (73% decrease) than adjuvant-only or nonimmune control animals. Alloantisera recognized more than 20 potentially unique sperm membrane proteins and five sperm lipid raft proteins resolved on two-dimensional immunoblots with or without prior enrichment by anion exchange chromatography. Dominant sperm membrane alloantigens identified by mass spectrometry included the ADAMs fertilin α, fertilin ß, and cyritestin. Less abundant alloantigens included ATP synthase F1 β subunit, myo-inositol monophosphatase-1, and zymogen granule membrane glycoprotein-2. Immunodominant sperm lipid raft alloantigens included SAMP14, lymphocyte antigen 6K, and the epididymal sperm protein E12. Of the fifteen unique membrane alloantigens identified, eleven were known sperm-specific proteins with uncertain functions in fertilization, and four were not previously suspected to exist as sperm-specific isoforms. *De novo* sequences of tryptic peptides from sperm membrane alloantigen “M6” displayed no evident homology to known proteins, so is a newly discovered sperm-specific gene product in swine. We conclude that alloimmunizing gilts with sperm membranes or lipid rafts evokes formation of antibodies to a relatively small number of dominant alloantigens that include known and novel sperm-specific proteins with possible functions in fertilization and potential utility as targets for immunocontraception.

## Introduction

Anti-fertility vaccines offer the promise of inexpensive, long-acting, non-hormonal control of reproduction in humans and in non-human pests from foxes to feral swine to elephants [1–4]. Pivotal early studies showed that vaccines directed against sperm antigens induce immunocontraception in small animals [5,6]. In large animals, however, inadequate knowledge of target antigens' immunogenicity and functions in fertilization have limited the effectiveness of sperm-based vaccines. Unique sets of sperm and egg proteins mediate the coordinated cellular events of fertilization [7,8]; many of these proteins are gamete specific, making them good candidate targets for immunocontraception. But unlike cellular and molecular processes in somatic tissues, fertilization events differ significantly among species [7], and individual sperm or egg proteins do not necessarily function identically in all species, especially if a process is mediated by gene family members with overlapping activities. Thus a full understanding of mammalian fertilization, and in turn the rational formulation of immunocontraceptives, will require characterization in multiple animal species of all sperm and egg proteins that mediate its various events.

Male germ cell-specific proteins induce auto- and alloimmune responses [3,5,9–11] modulated by regulatory T cells [12] upon egress of non-sequestered antigens from seminiferous tubules [13]. Sperm auto- and alloantibodies cause human infertility [14–15], and auto- and alloantibodies to whole sperm, to sperm fractions, or to individual sperm proteins can block various cellular events of fertilization *in vitro* [6,16–19]. Accordingly, anti-sperm auto- and alloantibodies have been used to identify sperm-specific proteins [11,19–23] that could serve as targets for contraception by blocking fertilization. Sperm-specific membrane proteins are potentially good targets for such contraceptives because they are accessible to antibodies or other inhibitors that can block the proteins' likely functions in essential membrane interactions such as gamete adhesion or exocytosis of the sperm acrosome. Likewise, membrane microdomains known as ‘lipid rafts’ or ‘detergent-resistant membranes’ (DRM) may contribute to the localization, organization, and regulation of specific signaling pathways [24–26] in spermatozoa of various species ranging from sea urchin to human [27–31], and thus may be an enriched source of rare sperm surface auto- or alloantigens with possible functions in fertilization.

Most studies of sperm auto- and alloantigens have been conducted using small mammals (mouse, rabbit, and guinea pig) [10,11,19–22] from which limited amounts of spermatozoa can be obtained for biochemical analyses. Consequently, no studies have yet systematically defined the full repertoire of sperm-specific membrane and lipid raft alloantigens in any single species. We previously showed that immunization of gilts (nulliparous female swine) with membrane fractions of boar spermatozoa induces alloimmunity to at least twelve potentially unique sperm membrane alloantigens (SMA) with possible functions in fertilization [3]. These SMA were predominantly acidic proteins localized to the plasma membrane overlying the entire head of boar spermatozoa, consistent with roles in early events of fertilization such as sperm capacitation, acrosome reaction, or zona pellucida interactions. Here, we tested whether alloimmunization with boar sperm membranes or lipid rafts affects fertility of gilts, and identified the major membrane and lipid raft alloantigens in this single species. We found that the relatively small number (10–20 proteins) of dominant boar SMAs and sperm lipid raft alloantigens (SLRAs) includes suitable targets for swine immunocontraception, and comprises both known sperm-specific proteins as well as one new protein not represented in existing protein sequence databases.

## Materials and methods

### Isolation of sperm plasma membranes and lipid rafts

Extended boar semen was generously provided by PIC USA (Hendersonville, TN) or obtained from the Texas Tech Swine Center (New Deal, TX). Spermatozoa recovered from semen by centrifugation (400 *g*, 8 min, 23°C) were washed once with phosphate-buffered saline (PBS; 10 mM NaPO_4_, pH 7.4, 150 mM NaCl) (Haden et al., 2000; Hickox et al., 2001; Bi et al, 2003) then resuspended at 4x10^8^ cells/ml in either HNE-DFP (10 mM HEPES pH 7.5, 140 mM NaCl, 1 mM EDTA, 0.5 mM diisopropylfluorophosphate (DFP)), or Tris- saline (TN; 5 mM Tris-HCl pH 7.4, 150 mM NaCl) containing 0.25 M sucrose (TNS) for plasma membrane isolation, or at 1x10^9^ cells/ml in HN (20 mM HEPES pH 7.5 130 mM NaCl) for lipid raft isolation.

Particulate fractions enriched in plasma membranes were isolated either as in our previous studies [3,32] ("Method 1") or by a potentially improved method [33] ("Method 2") with modifications (S1 Fig). We initially used Method 1 to prepare membranes for alloantiserum production (S2 Fig) and antigen identification, then switched to Method 2 because it yielded better enrichment in sperm plasma membranes and lower contamination by acrosomal proteins (S3 Fig). Membrane pellets were resuspended by Dounce homogenization either in HN or HKN (5 mM HEPES pH 7.5, 2.7 mM KCl, 146 mM NaCl), and washed three times with HN containing 1 M NaCl [3] to remove peripherally bound acrosomal proteins and increase the content of protein with a germ cell origin ("triple-washed membranes", TWM), resuspended in a minimal volume of HN, and stored at -70°C.

Lipid rafts in detergent extracts of boar spermatozoa were isolated by sucrose gradient ultracentrifugation [30]. Washed spermatozoa in HN were extracted with detergent by mixing with an equal volume of MN (50 mM MES pH 6.5, 150 mM NaCl) containing 1% Triton X-100 (Pierce Chemical Co., Rockford, IL) and protease inhibitors (Complete Mini EDTA-Free Protease Inhibitor Tablet, Roche Applied Science, Indianapolis, IN) and incubating the suspension for 20 min at 4°C. After removal of denuded spermatozoa by centrifugation (10 min, 900 *g*, 4°C), a detergent resistant fraction (detergent-resistant membranes, DRM) of the extract was resolved by sucrose gradient ultracentrifugation (200,000 *g*, 18 hours, 4°C, SW41 rotor, Beckman Instruments, Palo Alto, CA). DRM were detected by light scattering at 620 nm and by detection of the markers flotillin-2 and ganglioside G_M1_ (S4 Fig).

Protein concentration in TWM preparations and in sucrose gradient fractions was measured with bicinchoninic acid [34] (BCA Assay, Pierce Chemical Co.).

### Immunization of gilts and assessment of fertility

Gilts were alloimunized to pig sperm TWM or lipid rafts (S2 Fig) by intramuscular primary injection with 80 mg TWM protein (n = 4) or 10 mg raft protein (n = 2) per animal in Freund’s complete adjuvant [3], followed by intramuscular booster injections 6 weeks later with 80 mg TWM protein, or 10 mg raft protein in Freund’s incomplete adjuvant. Control animals (n = 4) were injected with adjuvant only. Sera (10 ml/animal) were collected 4 weeks after the single boost. On second estrus gilts were bred by artificial insemination (AI) with semen from boars of proven fertility, and the number of piglets born per female was determined at parturition.

This research was conducted according to animal use protocol approved by the Texas Tech University Animal Care and Use Committee, per provisions of the United States Department of Agriculture's Animal Care Policy Manual, Policy #17: Regulation of Agricultural Animals.

### Antigen enrichment by anion exchange chromatography

Our initial study detected SMA with mostly acidic pI [3]. Therefore, to scale up their isolation for subsequent identification we first enriched for acidic proteins by anion exchange chromatography. Briefly, TWM (500 mg protein) isolated by either method above were solubilized in 20 mM Bis-Tris-HCl pH 6.5 containing 50 mM NaCl and 2% reduced Triton-100 were fractionated by DEAE cellulose chromatography (HyTrap DEAE column, Amersham Biosciences, Uppsala, Sweden). Bound proteins were eluted at 1 ml/min with a linear 220 mM- 370 mM NaCl gradient, and fractions enriched in alloantigens with acidic pI were pooled for subsequent further separation by two-dimensional gel electrophoresis. Prior enrichment of acidic proteins in lipid rafts was not necessary because SLRA were present in our DRM preparations at sufficiently high levels for identification.

### Electrophoresis and immunoblotting

Proteins in sperm membrane and lipid raft preparations were resolved by SDS-PAGE (sodium dodecylsulfate polyacrylamide gel electrophoresis) on 12% acrylamide gels without disulfide bond reduction [3, 35]. To detect alloantigens, resolved proteins were transferred to polyvinylidene difluoride membranes (Immobilon P, Millipore Corp., Billerica, MA) [3,35], and blots were blocked for 1 h at 23°C with 2% (w/v) non-fat dry milk in TBST (10 mM Tris-HCl pH 7.5, 150 mM NaCl, 0.1% Tween 20) then probed by overnight incubation at 4°C with pooled alloantisera to TWM (designated "anti-TWM #1" [3], or "anti-TWM #2" = pooled sera from high responder gilts 466 and 474) or lipid raft proteins (designated "anti-rafts") diluted 1/5000 in 2% milk-TBST. Bound alloantibody was detected with horseradish peroxidase-conjugated protein A (Pierce Chemical Co.) diluted 1/10 000 in 2% milk-TBST, washed with TBST as above, and immunoreactivity revealed by enhanced chemiluminescence (Super Signal West Pico Chemiluminescent Substrate, Pierce Chemical Co., or Immun-Star WesternC Kit, Bio-Rad Laboratories, Hercules CA).

For two-dimensional gel electrophoresis of SMA and eventual western blotting, we resolved 50–100 μg sperm membrane protein by isoelectric focusing (IEF) on 7 cm Immobiline DryStrips (pH 4–7 or 3–10; GE Healthcare Bio-Sciences AB, Uppsala, Sweden) using a Multiphor II apparatus (Pharmacia LKB, Uppsala, Sweden) at 20°C for 1 min at 200 V, 20 min at 500 V, 20 min at 1000 V, 20 min at 1500 V, 20 min at 2000 V, 20 min at 2500 V and 1 h at 3000 V. In two-dimensional electrophoresis for SMA spot isolation and identification, we resolved 400–500 μg acidic protein fractions from anion exchange chromatography by isoelectric focusing on 18 cm strips for 2 h at 150 V, 1 h at 500 V, 1 h at 1000 V, 1 h at 2000 V, 1 h at 3000 V and 4 h at 3500 V. Lipid raft proteins (800–1500 μg from sucrose gradient fractions 4 and 5) were precipitated with 90% acetone at -20°C prior to isoelectric focusing on 18 cm strips as for SMA. Resolved proteins in the gel strips were then further separated in the second dimension by SDS-PAGE (12% acrylamide, disulfides not reduced), and detected by staining with BioSafe Coomassie or Silver Stain Plus (Bio-Rad Laboratories).

### Mass spectrometric identification of alloantigens

To identify SMA or SLRA we excised stained protein spots from preparative scale two-dimensional gels (18 cm Immobiline IEF strip, 15 cm second dimension), rinsed in deionized water, and in-gel digested with trypsin (proteomics grade no.T6567, Sigma Chemical Co., St. Louis, MO) [36]. Peptides released from the gel pieces were then purified using Zip Tip C18 micropipette tips (Millipore Corp.), co-crystallized with alpha cyano-4-hydroxy cinnamic acid on a standard stainless steel target, and analyzed in positive ion reflectron mode on a Shimadzu Biotech Axima TOF2 (Shimadzu Instruments, Carlsbad, CA) matrix-assisted-laser desorption/ionization Time-of-Flight (MALDI-TOF) mass spectrometer externally calibrated using a local spot to the sample of interest with Angiotensin II (1046.54 Da), P14R (1533.86 Da), and ACTH (18–39) 2465.20 Da. Post-Source-Decay (PSD) and Collisionally Induced Dissociation (CID) analyses were performed on the same instrument using a dual timed ion gate for high resolution precursor selection from the peptide mixture using a laser power about 20% higher than for MS acquisition. PSD and CID fragments were separated in a Curved Field Reflectron which allowed for a seamless full mass range acquisition of the MS/MS spectrum. All spectra were processed with Mascot Distiller (Matrix Science, Ltd., Boston, MA) prior to database searching.

Database searches were performed in-house with Mascot using the Peptide Mass Fingerprint program for MS searches (150 ppm peptide mass tolerance) and the MS/MS Ion Search program for MS/MS searching (PSD and CID spectra; 150 ppm precursor tolerance, 1 Da fragment tolerance). Variable modifications such as N-acetyl (protein), oxidation (M), Pyro-glu (N-terminal Q), and propionamide (C) were also considered, and a positive match indicated by a MOWSE score above the random match significance threshold (p<0.05), though definitive identification of a protein was not always limited to this criterion.

Some SMA and SLRA could not be positively identified by peptide mass analyses (MS and MS/MS) because the porcine genome was not fully sequenced and annotated. Consequently, when a peptide did not yield a positive match in Mascot searches, we determined its actual amino acid sequence by *de novo* MS/MS analysis. Tryptic peptides were derivatized with 4-sulphophenylisothiocyanate (SPITC) prior to analysis by MALDI-TOF-TOF-PSD to produce MS/MS spectra with simplified fragmentation patterns (y type ion series) that facilitate *de novo* sequence interpretation [37]. Derivatized peptides were *de novo* sequenced from manual analysis of their PSD spectra with the aid of the Mascot Distiller software, and their sequences compared by BLASTp (protein-protein BLAST) search of the NCBI nr mammalian database (algorithm parameters: expect threshold = 20,000, word size = 2, matrix adjustment = PAM30, compositional adjustment = none). Criteria for positive identification included expectation score (E value <20, and often <1), percentage identity, and similarity of protein size and pI to observed values from two dimensional electrophoresis.

### Statistical analyses

Data from the fertility trial (number of piglets born/sow) for the FCA-only and Immune groups (TWM high responders + rafts) were expressed as mean ± SEM and analyzed using the Student's t-test (n = 4) to assess the effect of immunization on litter size.

## Results

### Fertility of gilts immunized with sperm membranes or lipid rafts

**Fig 1:**
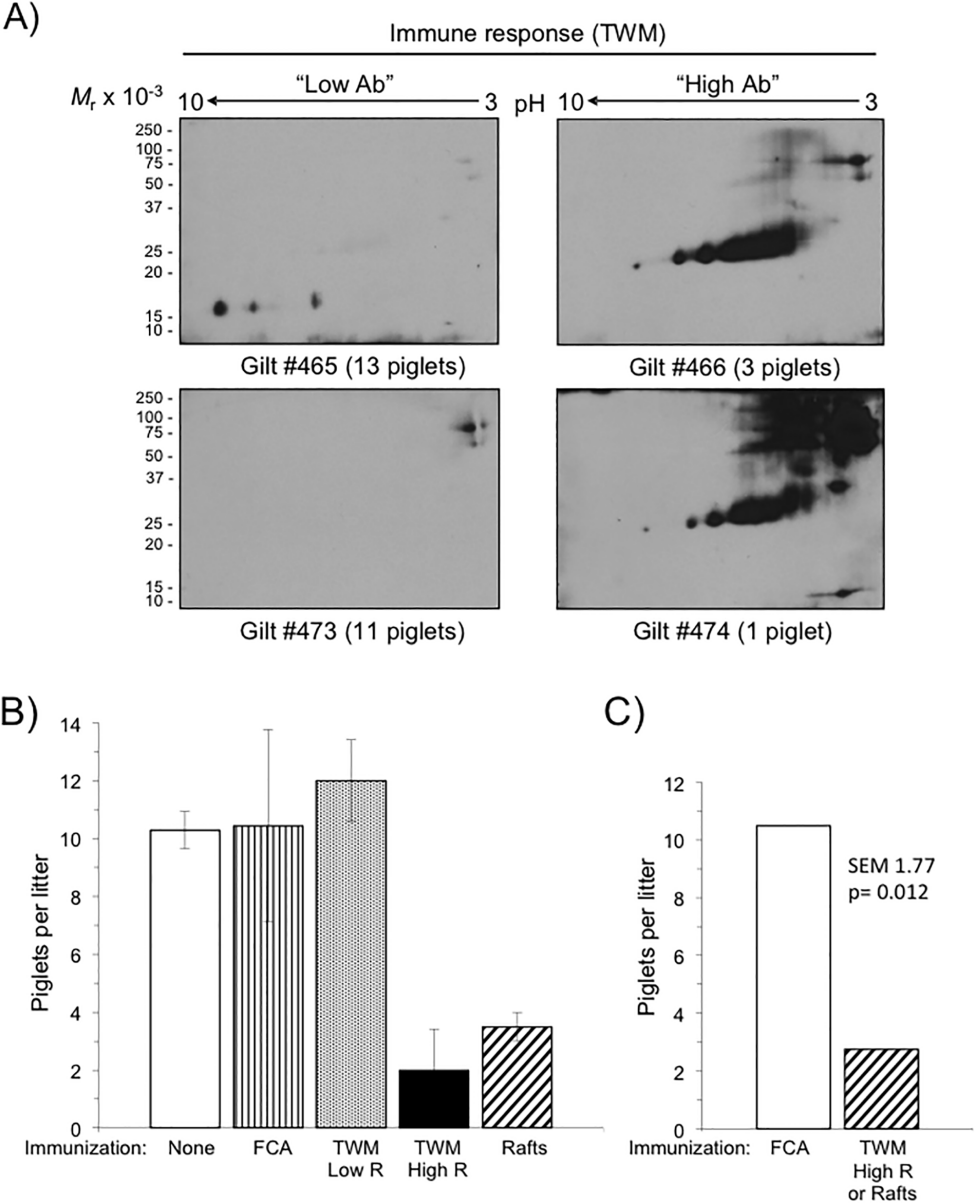
Fertility of gilts immunized with sperm membranes or lipid rafts. **A)** Detection of SMA with anti-TWM alloantisera from four different immunized gilts. Blots of TWM (triple-washed membranes, 50 μg protein/gel) resolved by 2-D gel electrophoresis were each probed with individual alloantisera (1/5000 dilution, sera collected 4 wk after boost), and immunoreactivity visualized with identical exposure times. Note the variability of the immune response evident as the high immunoreactivity of sera from two gilts (“High Ab,” #466 & 474), and lower immunoreactivity of two others (“Low Ab,” #465 & 473). **B)** Fertility of immunized gilts (TWM n = 4; lipid rafts n = 2) compared to gilts injected with adjuvant only ("FCA", n = 4). Gilts were bred on second estrus after collection of alloantisera used for the blots in Panel A. Data are expressed as mean litter size ± SD, with results for TWM-immunized high responder ("High Ab" blots in panel A) and low responder animals shown separately. For reference, shown also is our swine farm’s average litter size for non-immunized sows ("None") over a preceding 7-month period (2528 offspring/247 litters = 10.3 ± 0.6). **C)** Effect of alloimmunization on fertility. We compared litter sizes of high responder gilts immunized with TWM or lipid rafts to litter sizes of gilts injected with Freund's Complete Adjuvant only ("FCA"). Shown are mean litter sizes and standard error of the means with n = 4 in each group. High immunity to SMA or SLRA produced a 3.8-fold decrease in litter size (from 10.5 to 3.3 piglets/litter, *p* = 0.0119).

Immunization with sperm ‘triple-washed membranes’ (TWM) or lipid rafts evoked alloimmune responses to numerous sperm proteins (Fig 1). To assess the effect of immunization on fertilization *in vivo*, gilts were bred by AI on second estrus with semen from boars of proven fertility. Injection with sperm TWM elicited a variable immune response (Fig 1A), so immunized gilts were grouped as either high responders (gilts #466 and 474) or low responders (gilts #465 and 473) for statistical analysis. Litter sizes of the adjuvant only control group ("FCA", Fig 1B; 10.5 ± 3.3 piglets/ litter, n = 4) were nearly identical to the long term, month-by-month average (10.3 ± 0.6 piglets/litter) for non-immunized sows in our swine farm (247 litters over the preceding 7 months) and to the TWM low responders (12.0 ± 1.4 piglets/litter, n = 2) did not differ (p> 0.05) from litter sizes of adjuvant only control gilts ("TWM-low Ab" vs. "FCA"; Fig 1B). In contrast, high responder gilts immunized with either TWM or lipid raft proteins (n = 4) gave birth to fewer piglets/litter (2.8 ± 1.3) as compared to the adjuvant only control (p = 0.0119; Fig 1C), representing a 73% decrease in fertility.

### Detection and isolation of boar sperm membrane alloantigens (SMA)

**Fig 2:**
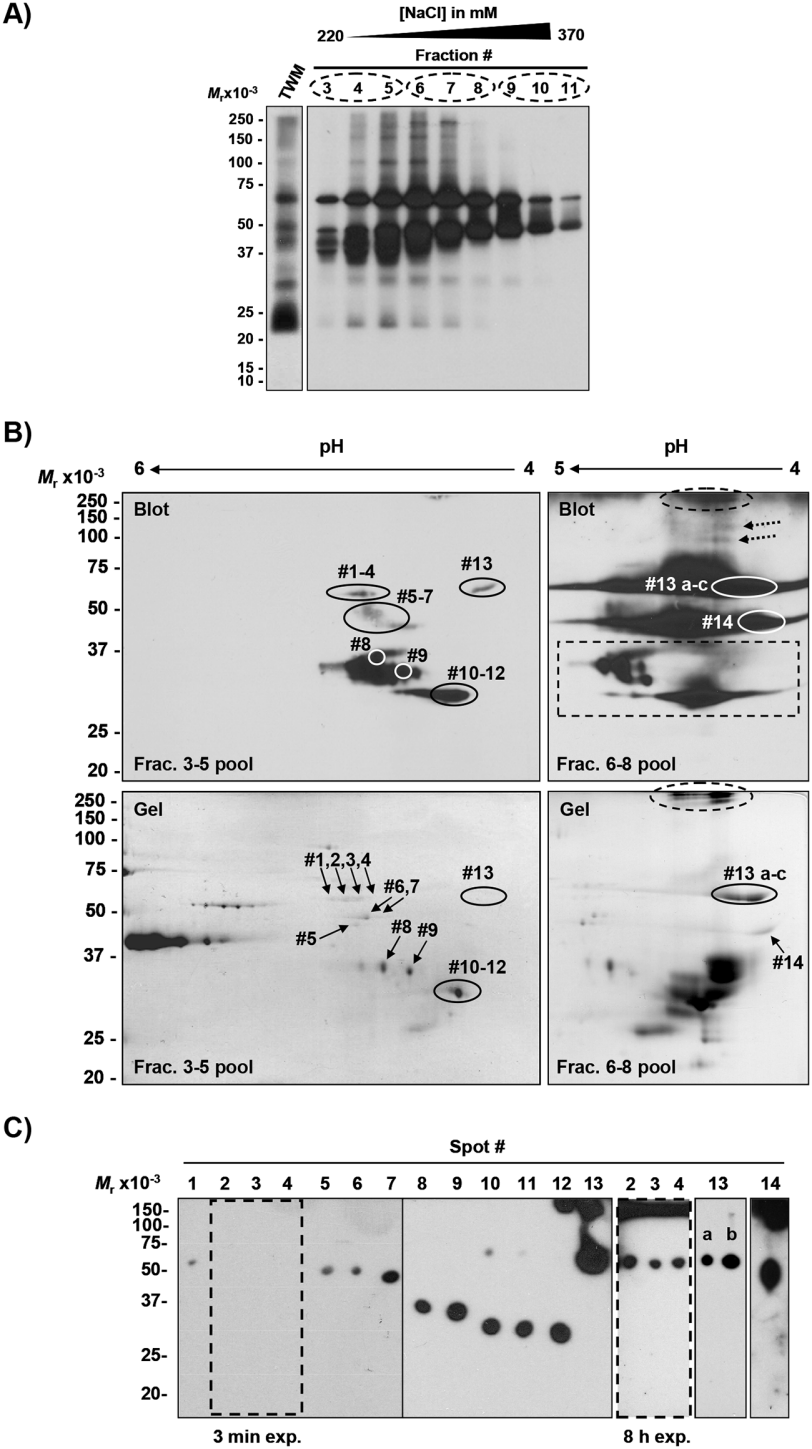
Detection of acidic SMA in membranes isolated by Method 1 on western blots probed with sperm membrane alloantisera pool anti-TWM #1. **A)** Pre-enrichment of acidic SMA by anion exchange chromatography. **B)** 2-D electrophoretic profiles of acidic immunoreactive SMA eluted with 220–260 mM NaCl (fractions 3–5 pool), left panels, and 280–310 mM NaCl (fractions 6–8 pool), right panels. Proteins from sperm particulate fraction containing released plasma membranes were isolated by Method 1 as described in “Materials and Methods”. Following pre-enrichment, acidic SMA eluted with a continuous NaCl gradient were solubilized in solution containing 9.8 M urea (disulfides not reduced), loaded onto 18 cm IPG strip (pH 4–7), and resolved by IEF followed by SDS-PAGE. Immunoreactive SMA were detected by western blotting (top panels) using anti-TWM #1 (1/5000 dilution of pooled sera from [3]), and all SMA were visualized with Coomassie blue (bottom panels). Immunodominant SMA were cored from duplicate preparative gels and analyzed by MS. Two high-molecular weight SMA with M_*r*_ 100,000 and 150,000 (dotted arrows) not detected on the corresponding 2-D gel (right panels) were not analyzed by MS. Likewise, the analysis of SMA with a M_*r*_ ≥ 250,000 (*circled* in dashed line) was not pursued. SMA #8–12 (*boxed* in dashed line) were previously detected in fractions 3–5 pool (left panel) and were not reanalyzed. **C)** Confirmation of immunoreactivity of individual spots cored from 2-D gel as shown in **B)**.

**Fig 3:**
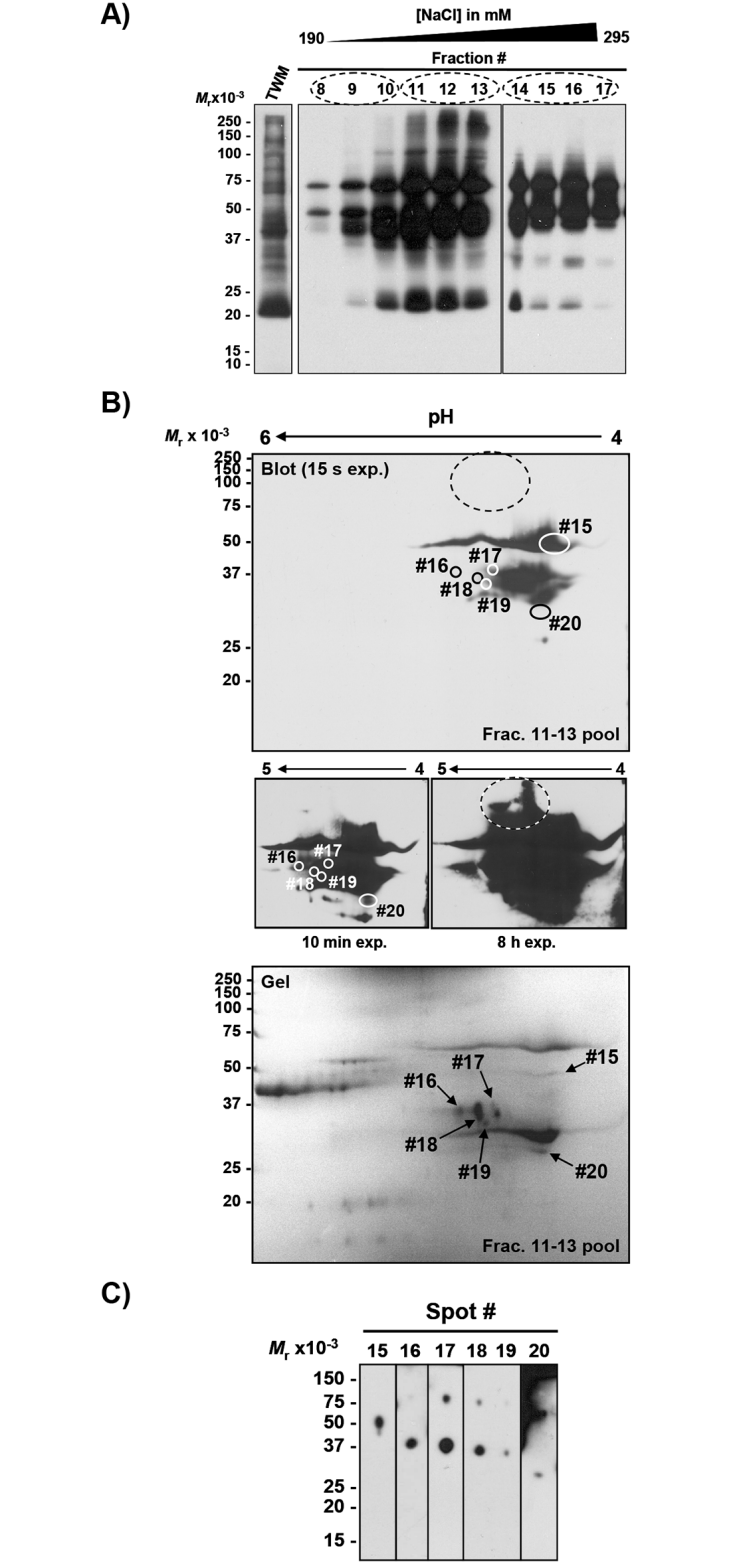
Detection of acidic SMA in membranes isolated by Method 2 on western blots probed with sperm membrane alloantisera pool anti-TWM #1. **A)** Pre-enrichment of acidic SMA by anion exchange chromatography. **B)** Profile of acidic immunoreactive SMA eluted with 240–280 mM NaCl. Acidic SMA eluted with a continuous NaCl gradient were solubilized (disulfides not reduced) and resolved by IEF(pH 4–7)/SDS-PAGE as for Fig 2. Immunoreactive SMA were detected by western blotting (top panel) using anti-TWM #1 (1/5000 dilution of pooled sera from [3]), and all SMA visualized with Coomassie blue (bottom panel). Immunodominant SMA were cored from duplicate preparative gels and analyzed by MS. Five SMA (#8–12) previously immunodetected in fractions 3–5 pool (Fig 2B) were not labeled on the blot. Immunoreactive SMA with M_*r*_ 100,000–250,000 (*circled* in dashed line) not visualized on the corresponding 2-D gel were not analyzed further. **C)** Confirmation of individual spot’s immunoreactivity previously resolved on 2-D gel as shown in B).

We first isolated and characterized dominant alloantigens recognized by pooled alloantisera from a previous study [3], designated anti-TWM #1. These antisera primarily detected proteins with acidic pI [3], so to maximize yield from two-dimensional electrophoresis we first pre-enriched acidic SMA by anion exchange chromatography (Figs 2A and 3A). Most of the acidic SMA eluted at relatively low [NaCl] (220–260 mM NaCl; fractions 3–5) for the Triton-solubilized extract of TWM isolated using Method 1 (Fig 2A), or 240–280 mM NaCl (fractions 11–13) for the TWM isolated using Method 2 (Fig 3A). Both purifications resulted in enrichment of acidic SMA with M_*r*_ ranging from 35,000–250,000 detected with anti-TWM #1. Three immunoreactive bands containing SMA of M_*r*_ 100,000–250,000 that were not observed in the initial TWM Triton-extract (Method 1) were detected in the enriched fractions 5–7 (Fig 2A). Similarly, another polypeptide with M_*r*_ 37,000 was only detected in fractions 3–5. Prior to DEAE fractionation, TWM Triton-extract from Method 2 already exhibited a different profile of immunoreactive SMA (Fig 3A). Nevertheless, enrichment of five acidic SMA was apparent by the enhancement of immunoreactivity at M_*r*_ 35,000, 50,000, 60,000, 100,000, and 250,000.

Anti-TWM #1 detected numerous acidic SMA on western blots among the restricted profile of proteins in pools of eluted fractions resolved by two-dimensional electrophoresis (Figs 2B and 3B). Thirteen immunoreactive SMA from Triton-solubilized extract of TWM isolated using Method 1 (pooled fractions 3–5) were visualized on two- dimensional immunoblot (Fig 2B). Only one additional SMA (SMA #14) was detected in pooled fractions 6–8 (right panel); the other immunoreactive SMA were already identified as SMA #8, 9, 10–12, and 13 on the 2-D blot of pooled fractions 3–5 (left panel). Two high-molecular weight SMA with M_*r*_ 100,000 and 150,000 were detected only in fractions 6–8 (dotted arrows, Fig 2B, upper right panel) were not visible on the duplicate Coomassie-stained two-dimensional gel, so their identification by MS was not feasible with the amount of material available. Very large SMA (M_*r*_ ≥250,000) did not resolve on the 12% SDS-PAGE as revealed by the proteins stacked at both the top of the gel and blot between pIs 4.3–4.6 (dashed oval, Fig 2B, upper right panel), so also were not analyzed further. Two-dimensional analysis of the remaining eluate (pooled fractions 9–11) did not detect any new SMA on immunoblot, confirming that most of the acidic immunoreactive SMA were eluted with low [NaCl].

Anti-TWM #1 recognized six additional SMA in pooled fractions 11–13 of TWM isolated using Method 2 and subsequently enriched by anion exchange chromatography (Fig 3B), thus confirming the utility of pre-enriching for acidic proteins prior to SMA detection and identification. As previously observed, acidic SMA with M_*r*_ ranging from 100,000–250,000 (dashed ovals, Fig 3B) could not be visualized on Coomassie-stained two-dimensional gel, and the corresponding blot required a 45-fold longer exposure to detect them, so their identification was not feasible with the amount of material available. Two-dimensional gel/blot analysis of fractions pooled 8–10 and 14–17 detected no new SMAs, and no spots were cored from those gels.

One-dimensional gel/blot analysis of individual gel spots cored from duplicate preparative two-dimensional gels confirmed that the desired SMAs had been successfully recovered (Figs 2C and 3C). The films from the short exposures were also used to estimate the M_*r*_'s of immunoreactive SMA, and their M_*r*_'s were similar to those previously observed on two-dimensional gels (Figs 2B and 3B).

### Mass spectrometric identification of dominant SMA

After prior enrichment by anion exchange chromatography, we obtained acidic, immunodominant SMA in sufficient quantity to identify them by peptide mass fingerprinting, MS/MS ion search, and BLASTp comparison of *de novo* sequences with the NCBI non-redundant (nr) mammalian protein database (Table 1). The 20 potentially unique SMA detected on six different two-dimensional blots derived from fourteen unique proteins, either because: 1) the same SMA was detected in multiple spots on the same blot; 2) the same SMA was detected on multiple blots; 3) immunoreactive SMA were not visible on the corresponding two-dimensional gel; 4) more than one SMA was identified in a single gel spot; or 5) a SMA resolved as a train of proteins and only one of them was analyzed by MS. For SMA spots 8–18 no swine protein entries were present in the NCBI NR database, so they were not identifiable by mass search. We therefore identified them by similarity to non-swine orthologs in BLAST searches of their *de novo* sequences (Table 1). While this manuscript was in preparation, the Swine Genome Sequencing Consortium contributed a large number of annotated entries to the NR database, enabling positive confirmation of our initial identifications by both mass and sequence comparison to the predicted swine proteins.

**Table 1:** Identification of SMA detected with anti-TWM #1.

Spot	M/Z (+1)	Peptide sequence	I.D. (Accession #)	Remark
2	1405.65	R.MGLYPGVLEPSSR.G	Arylsulfatase A(AAL58668)	Five peptides shown of 12 total
	1708.72	K.AQFDAAVTFSPSQIAR.G		
	1737.92	R.GGLPLEEVTLAEVLAAR.G		
	2238.95	K.WHLGVGPEGAFLPPHQGFHR.F		
	3483.17	R.QGRPFFLYYAASHHTHYPQFSGQSFSGHSGR.G		
5	1406.56	K.AHGGYSVFAGVGER.T	ATP synthase F1 β subunit (CAA29094; XP_001929445)	All peptides homologous to β subunit of *B*. *taurus* heart ATP synthase F1 and confirmed identical to predicted *S*. *scrofa* protein
	1601.68	K.VALVYGQMNEPPGAR.A		
	1815.74	R.IMDPNIVGSEHYDVAR.G		
5–7	1987.97	R.AIAELGIYPAVDPLDSTSR.I	ATP synthase F1 β subunit (2F43_B; AAB02288; XP_001929445)	Three peptides shown of 20 total, all homologous to β subunit of *R*. *norvegicus* liver F1 ATPase and confirmed identical to predicted *S*. *scrofa* protein
	2318.07	R.TREGNDLYHEMIESGVINLK.D		
	3843.04	K.KGSITSVQAIYVPADDLTDPAPAT		
		TFAHLDATTVLSR.A		
6	1262.68	R.TIAMDGTEGLVR.G	ATP synthase F1 β subunit (ABD77251)	Three peptides shown of 40 total, all matching β subunit of *S*. *scrofa* liver mitochondrial ATPase/synthase, H^+^ transporting F1 complex
	2005.03	R.FLSQPFQVAEVFTGHLGK.L		
	2266.01	R.IPSAVGYQPTLATDMGTMQER.I		
*8	1342.61	NFDTQYTYYK	Cyritestin, or ADAM 3 (XP_532794)	Homologous to a disintegrin and metalloprotease domain 3 (cyritestin) of *C*. *familiaris*
	2129.12	(SE or TD)VVPFKNFDTQYTYYK		
*9,*13	1178.57	FGNCGXTTXPR	Fertilin α, or ADAM 1 (CAA75659)	One peptide homologous to fertilin α of *P*. *pygmaeus*
	1712.77	SDDXQSEDVGGGGVLQH		
*10,	1600.72	TDESGACGXTASGYXR	Fertilin β, or ADAM 2 (CAC84225)	Confirmed by mass fingerprint and MS/MS ion searches of 10 additional peptides from spot cored from another gel.
*12				
*15,	1235.67	FGGGCGXTTXPR	Fertilin α, or ADAM 1 (CAA75659; CAA56203; CAA56204)	Two peptides homologous to *P*. *pygmaeus* fertilin α and one to fertilin α isoforms I and II of various species including *M*. *fascicularis*
*19	1441.67	SAPENCYXSMNR		
	1868.8	SDDLQSEDVGGGGVXQHR		
*14	975.57	VVCTNVQR	Fertilin α, or ADAM 1 (CAA56203)	One peptide homologous to fertilin α of *M*. *fascicularis*
	1082.56	NFCVGGXCR		
	1871.9	SFDYQCFDVFGYPAR		
*14	1269.69	XSCVHTSPVNR	Sperm acrosome membrane associated protein 1, SAMP1 (XP_854415; NP_112222)	Homologous to *C*. *familiaris* sperm acrosome membrane-associated 1 and *H*. *sapiens* SAMP1 precursor (SAMP32)
*16,	1116.63	GXXCVSAQXR	Cyritestin, or ADAM 3b (XP_532794)	Homologous to *C*. *familiaris* a disintegrin and metalloprotease domain 3 (cyritestin)
*17	1259.52	TAFCFQGXCR		
	2323.14	G(AST)XCSQHVDGQTDDFGN(SAR)		
*18	1056.42	FCDNGQCR	Cyritestin, or ADAM 3b (CAA54085)	One peptide homologous to cyritestin of *M*. *fascicularis*
	1305.57	TTYCFQGLCR		
*20	1191.63	R.LLCIPIHGIR.G +C	*myo*-inositol monophosphatase (NP_999381; NP_776786)	Four peptides shown of 17 total, all matching *S*. *scrofa myo*-inositol monophosphatase (tissue not specified).
	1583.66	K.EIQIIPLQRDDED.-¶		
	2064.87	K.EKYPSHSFIGEESVAAGEK.S¶		
	2371.16	K.LQVSPQKDVTNSLLVTELGSSR.T		

X = isoleucine (I) or leucine (L)

* de novo sequencing was performed

Peptide modifications: +C, carbamidomethyl

^¶^ Also homologous to *B*. *taurus* brain enzyme

We identified the major SMA's as the ADAMs fertilin α (Adam1), fertilin β (Adam2), and cyritestin (Adam3). The alloantisera recognized only mature, processed forms of ADAM proteins in Triton-solubilized extracts of sperm TW, as indicated by the lower experimental M_*r*_'s of these acidic SMA on SDS-PAGE (protein disulfides not reduced) when compared to their M_*r*_'s calculated from the deduced amino acid sequence of the protein precursor (Table 2). For instance, we noted a difference of M_*r*_ 39,000 between the actual and predicted sizes of ADAM3 (spots #8, 16 & 17), and a difference of M_*r*_ 50,000 for ADAM2. Our sperm membrane preparations contained several ADAM isoforms as revealed by their differing M_*r*_'s and pI's on separation by two-dimensional gel electrophoresis (disulfides not reduced). Though adventitious proteolysis could also produce such heterogeneity, TWM preparation Method 1 and Method 2 both included protease inhibitors, and we did not observe small immunoreactive peptides that would be produced by such proteolysis.

**Table 2:** Properties of SMA detected with anti-TWM #1.

Spot	I.D.	Mr,/pI		Putative function [Reference]
		Theoretical*	Experimental**	
2	Arylsulfatase A	56,603/5.37	54,000/4.86	Adhesion to ZP; digestion of cumulus cells [38,39]
5	ATP synthase F1 β subunit	51,353/4.95†	48,000/4.84	Motility; acrosome reaction [40]
6	ATP synthase F1 β subunit	47,089/4.99	51,800/4.78	
7	ATP synthase F1 β subunit	51,202/4.92†	51,800/4.62	
8	Cyritestin/ADAM 3	83,583/8.25	36,000/4.60	Adhesion to ZP, sperm migration in oviduct [41]
9	Fertilin α/ADAM 1	94,446/5.40	34,000/4.52	Transport of sperm proteins including ADAM3; sperm migration into oviduct [41]
10, 12	Fertilin β/ADAM 2	81,805/5.53	30,700/4.41	Adhesion to ZP, sperm migration into oviduct [41]
13	Fertilin α/ADAM 1	94,446/5.40	60,000/4.40	ADAM1a/ADAM2, transport of sperm proteins, including ADAM3 [41]; ADAM1b, Unknown
14	Fertilin α/ADAM 1	94,446/5.40	40,000/4.54	
14	Sperm acrosome membrane-associated protein 1, or SAMP1	39,782/5.42	40,000/4.54	Egg adhesion; fusion and sperm internalization [42]
15	Fertilin α/ADAM 1	94,446/5.40	53,000/4.54	ADAM1a/ADAM2, transport of sperm proteins, including ADAM3 [41]; ADAM1b, Unknown
16	Cyritestin/ADAM 3	83,583/8.25	36,000/4.88	Adhesion to ZP, sperm migration in oviduct [41]
17	Cyritestin/ADAM 3	83,583/8.25	36,000/4.75	
18	Cyritestin/ADAM 3	83,583/8.25	34,000/4.80	
19	Fertilin α/ADAM 1	94,446/5.40	33,000/4.77	ADAM1a/ADAM2, transport of sperm proteins, including ADAM3[41]; ADAM1b, Unknown
20	myo-inositol monophosphatase	30,133/4.92	26,600/4.53	Conversion of inositol-1 phosphate to myo-inositol [43]

*Computed from sequence data from *Sus scrofa* where available, using Primary Structure Analysis Tools of Swiss Institute of Bioinformatics ExPASy Proteomics Server, or EditSeq (Lasergene)

**Protein disulfides not reduced

^†^Calculated using sequence data from *Rattus norvegicus*

MS analysis of boar SMA also identified the previously characterized sperm proteins arylsulfatase A and sperm acrosome membrane-associated protein 1 (AS-A and SAMP1; Table 1). For both proteins, deduced and experimental M_*r*_ were very similar to each other (Table 2), but different from reported experimental values. AS-A was first identified in boar spermatozoa as 'P68' based on its migration with M_*r*_ 68,000 in SDS-PAGE (protein disulfides reduced), and SAMP1, only characterized in human, with M_*r*_ of 32,000 (SAMP32). In our two-dimensional electrophoresis, AS-A resolved as a train of four M_*r*_ 54,000 SMA's (Fig 2B left panels: spots #1–4, spot #2 analyzed by MS); we considered it a single SMA because such an electrophoretic pattern is characteristic of heterogeneous post-translational modifications. In contrast, SAMP1 resolved as a single M_*r*_ 40,000 spot (#14, Fig 2B right panels), that also contained a second polypeptide identified as ADAM1 (Table 1).

We also identified ATP synthase F1 ß subunit and myo-inositol monophosphatase-1 as minor alloantigens of the membrane-enriched particulate fraction. For both of these SMAs, experimental values for M_*r*_ and pI agreed well with deduced values, although their peptide masses matched with proteins from heart, liver and brain (Table 2).

### Detection and identification of additional SMA

**Fig 4:**
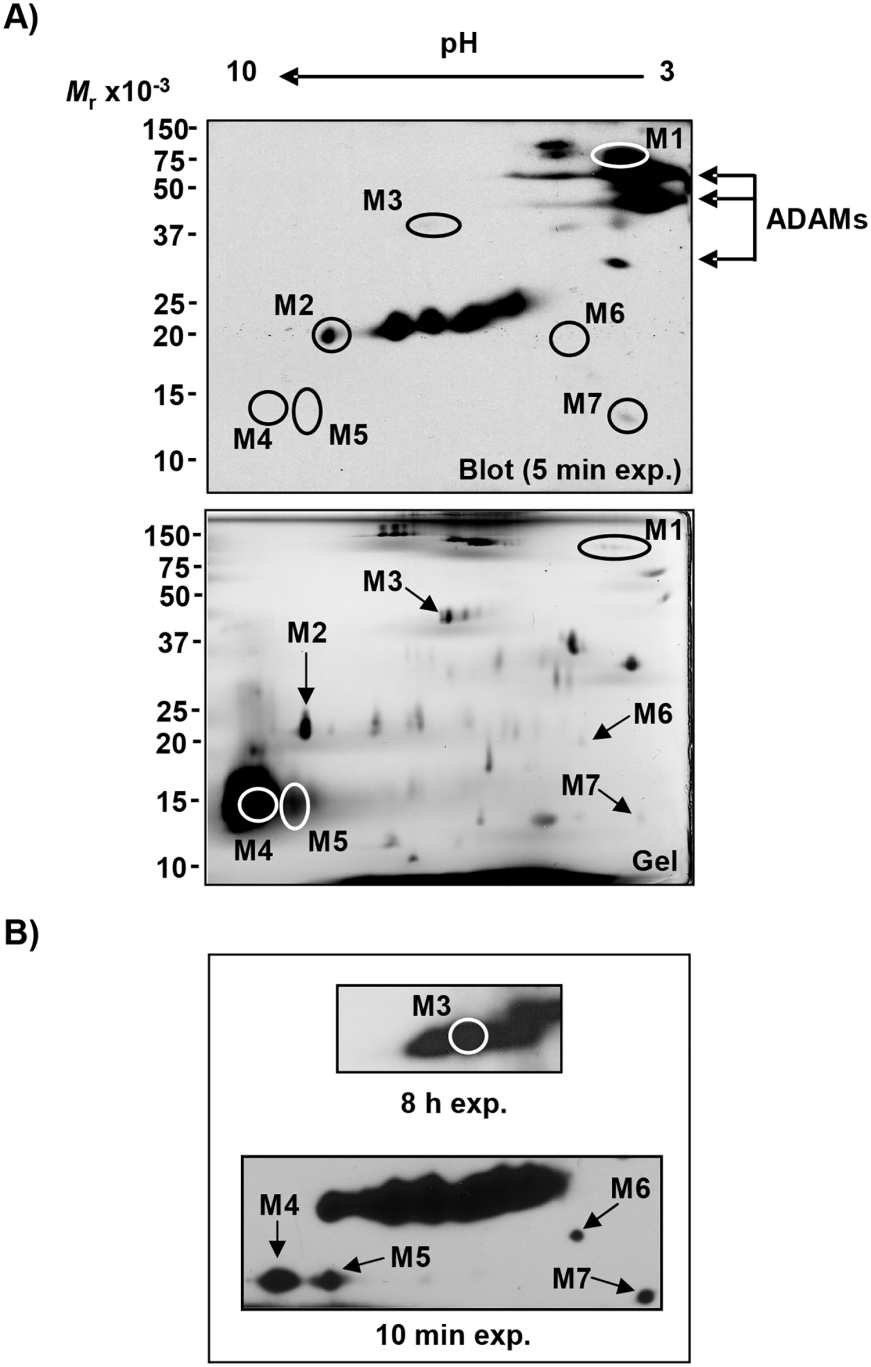
Detection of SMA by 2-D western blotting with new alloantisera to sperm membranes (anti-TWM #2). **A)** Immunoreactive SMA detected by western blotting (top panel) using anti-TWM #2 (1/5000 dilution of pooled high responder sera) and visualization of all SMA with silver stain (bottom panel). Particulate fractions enriched in plasma membranes of boar spermatozoa were isolated by Method 2 as described in “Materials and Methods”. TWM proteins were solubilized (disulfides not reduced) and resolved by IEF (pH4-7)/SDS-PAGE as for Figs 2 and 3, and SMA detected by Western blotting with anti-TWM #2. Immunodominant SMA (except M5) were cored from duplicate gel, and analyzed by MS. Black arrows indicate acidic SMA previously identified as ADAMs (Fig 2B). **B)** A longer exposure of the blot shown in A) allowed the immunodetection of SMA M3-M7.

To extend antigen identification results obtained with anti-TWM #1 alloantisera, we similarly detected alloantigens using new alloantisera to more highly purified sperm membranes (anti-TWM #2; high responder pool). Without antigen pre-enrichment, anti-TWM #2 confirmed the alloantigenicity of proteins identified by anti-TWM #1, and recognized seven additional SMA spots on two-dimensional immunoblot (Fig 4, top panel). Anti-TWM #2 detected ADAMs at M_*r*_ 30–60,000 and pI 3.0–4.5 as previously identified by anti-TWM #1. Peptide MS analysis of other spots identified five more SMA, including the known sperm proteins SP47, sperm acrosomal membrane protein 14 (SAMP14), and spermadhesins AWN-1 and AQN-3 (Table 3). The spermadhesins and DQH sperm surface protein migrated as a single spot (M4) that stained heavily with silver (Fig 4, bottom panel). Spot M5 seemed to represent a variant of the SMA identified in spot M4 (same 15,000 M_*r*_ with a slightly more basic pI), suggesting it also comprised spermadhesins, so MS analysis of M5 was not pursued.

**Table 3:** Identification of SMA detected with anti-TWM #2.

Spot	M/Z (+1)	Peptide sequence	*S*. *scrofa* I.D. (Accession no.)	Remark
*M1	711.39	YFXXR	Pancreatic secretory granule membrane major glycoprotein GP2 isoform 1 (XP_005662159)	Homologous to GP-2 isoform 1 of various species [e.g. *M*. *mulatta* (EHH31479) *H*. *sapiens* (NP_001007241)]
	1210.63	ACQGGYHVYR		
	1217.59	XESTPQCNXR		
M2	966.45	R.AVYDGQWK.Y	Epididymal sperm binding protein E12 precursor (CAD62255)	Four smallest peptides also homologous to *S*. *scrofa* epididymal sperm binding 1 precursor (NP_999569)
	1138.52	K.GFTYFSCTR.T +C		
	1228.58	K.YCLIEDYPR +C		
	1292.68	R.TNSLSPWCATR.A +C		
	1620.85	G.DTKDSCVFPFNYK.G +C		
M3	1105.60	R.ILPVAWHNR.I	Sperm surface protein SP47, or lactadherin (P79385)	
	1271.57	K.NMFETPFLTR.F +M		
	1537.77	R.AGIVNAWTASNYDR.N		
	1647.77	K.VAYSDDGVSWTEYR.D		
	2446.17	K.VAYSDDGVSWTEYRDQGALEGK.I		
M4	903.60	R.QTIIATEK.N	Spermadhesin AWNA1 (AAB21990)	
	1005.59	K.IFNSDGPQK.D		
	1242.75	R.SCGGVLRDPPGK.I +C		
	808.45	K.EYLEVR.D	Seminal plasma sperm motility inhibitor precursor; spermadhesin AQNA3 (NP_001026946)	
	861.52	R.AGPDNFLK.V		
	2005.01	K.VCGGTTFVYQSSSNVATVK.Y +C		
	822.49	.DQHLPGR.F	Seminal plasma protein pB1; DQH sperm surface protein (P80964)	
	1713.78	K.YWCPVTPNYDQDR.A +C		
*M6	1224.56	WEEAYXNSGR	None	No evident homology to swine sequences
	1553.80	NSKWEEAYXNSGR		
*M7	1137.58	GCVQSTXCGR	Sperm acrosome membrane-associated protein 4 precursor (NP_001171400)	Homologous to *M*. *musculus* and *H*. *sapiens* SAMP4 (NP_081331, NP_598005)

X = isoleucine (I) or leucine (L)

*de novo sequencing was performed

Peptide modifications: +C, carbamidomethyl, +M, oxidation

The major SMA's recognized by anti-TWM #2 included one of epididymal origin, the epididymal sperm binding protein E12 (ELSPBP1). We analyzed one ELSPBP1 gel spot (M2) by MS among a train of strongly immunoreactive spots with similar M_*r*_ (20,000–25,000) spanning a range of pI from 4.5 to 8.0 (Fig 4). This pattern likely reflected several ELSPBP1 charge isoforms as shown for the human ELSPBP1 ortholog, so MS analysis ceased with spot M2.

Similar to our identification of SMA detected by anti-TWM #1, three SMA detected by anti-TWM #2 (M1, M6, M7) were not identifiable by mass analysis, so were instead identified by BLAST search for orthologous sequences. We thus identified the minor SMA “M1”, that resolved as a train of five spots in two-dimensional electrophoresis, as the major component of zymogen granule membrane glycoprotein 2 (GP-2) of the exocrine pancreas (Table 3). The deduced mass (59,480 kDa) and M_*r*_ (58,000; protein disulfides reduced) reported in the literature for pancreatic GP-2 both differed from the M_*r*_ 125,000 we observed for the sperm GP-2 (Table 4), suggesting the polypeptide recognized by our alloantisera is a sperm-specific variant distinct from the pancreatic form.

**Table 4:** Properties of SMA detected with anti-TWM #2.

Spot #	I.D.	Mr,/pI		Putative function [Reference]
		Theoretical*	Experimental**	
M1	Zymogen granule membrane glyco-protein 2 precursor/GP-2	59,480/5.08†	125,000/3.98	Protein secretion; ion transport [44]
M2	E12 precursor/Epididymal sperm binding protein 1/ELSPBP1	26,200/7.8	22,000/8.14	Sperm-egg adhesion; capacitation [45,46]
M3	Lactadherin/Sperm surface protein SP47	45,725/6.15	43,000/6.06	Adhesion to ZP [47]
M4	Spermadhesin AWN-1	14,776/9.33	15,000/8.86	Adhesion to ZP; binding to oviductal cells [48–52]
	Spermadhesin AQN-3	15,034/9.06		
	DQH sperm surface protein	12,707/8.42		
M6	Various hypothetical proteins	Various	20,000/4.30	None known
M7	Sperm acrosome associated 4/ SAMP4/sperm acrosomal mem-brane protein 14/SAMP14	13,004/5.49†	13,000/3.43	Egg adhesion and fusion [53]

*Computed from sequence data from *Sus scrofa* where available, using Primary Structure Analysis Tools of Swiss Institute of Bioinformatics ExPASy Proteomics Server, or EditSeq (Lasergene)

**Protein disulfides not reduced

^†^Calculated using sequence data from *Homo sapiens*

Two peptides from *de novo* sequencing of the minor sperm alloantigen SMA “M6” at M_*r*_ 20,000 and pI 4.30 initially yielded no significant matches in BLASTp comparisons to known sequences using the NCBI nr mammalian database (Table 3).

### Identification of boar sperm lipid raft alloantigens (SLRA)

**Fig 5:**
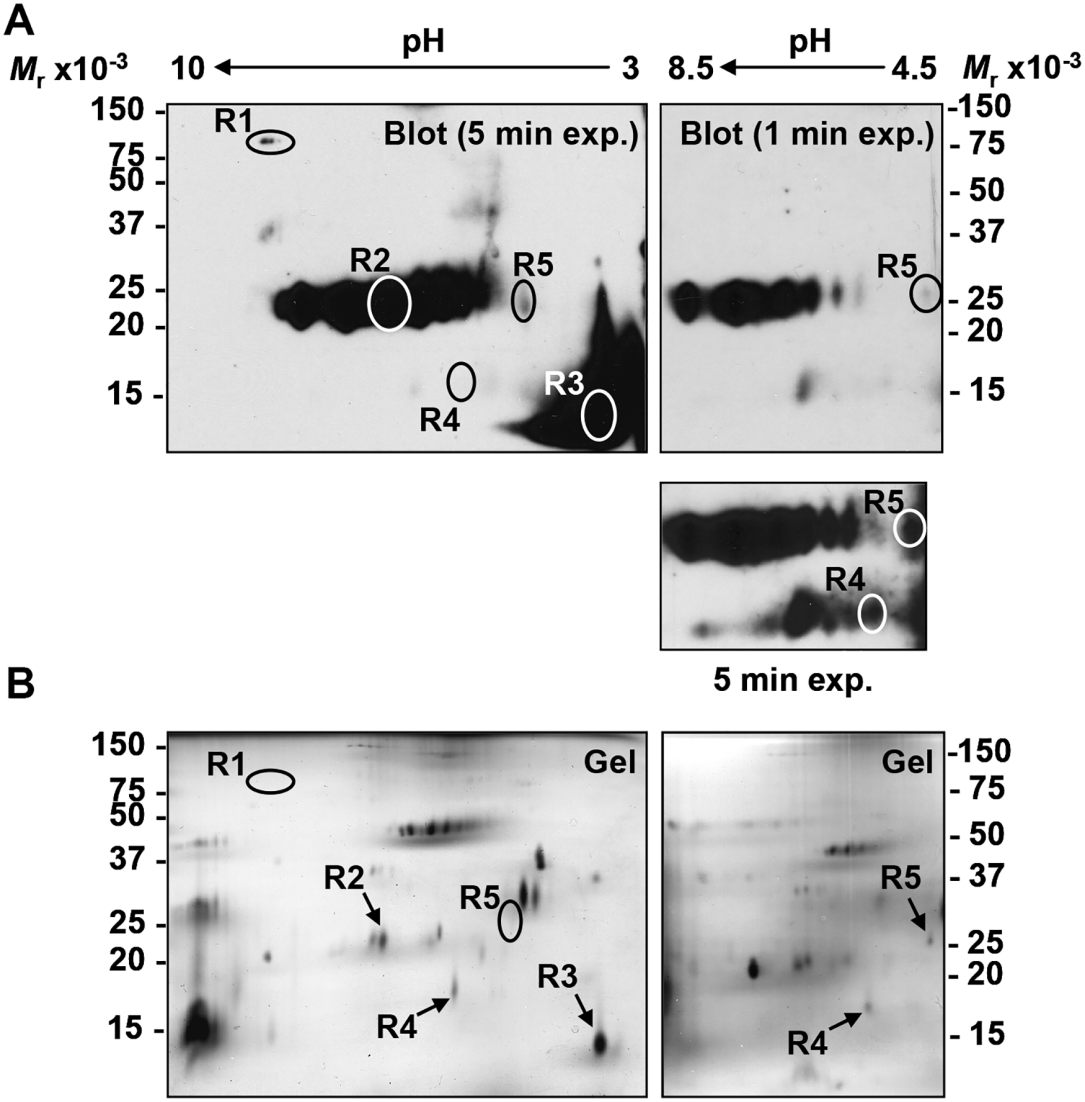
Detection of SLRA by 2-D western blotting with alloantisera to raft proteins (anti-rafts). **A)** Profile of immunoreactive SLRA detected by western blotting using anti-rafts alloantisera (pool from gilts 470 and 471). **B)** Visualization of all raft proteins with silver stain. Lipid rafts in detergent extracts of boar spermatozoa were isolated by sucrose gradient ultracentrifugation. Raft proteins, fraction 4 as determined by light-scattering at 620 nm and the presence of flotillin-2, were precipitated with acetone (A and B, left panels). Alternatively, fractions 4 and 5 were pooled and ultracentrifuged (A and B, right panels). Raft proteins were solubilized in rehydration solution containing 9.8 M urea (disulfides not reduced), resolved by IEF/SDS-PAGE, and SLRA were detected by Western blotting (1/5000 dilution of pooled sera from gilts #470 and #471). Immunodominant SLRA were cored from a duplicate gel for analysis by MS.

Alloantisera to lipid rafts (anti-rafts) recognized five immunoreactive SLRA on two-dimensional immunoblot (Fig 5A). One SLRA (“R1”, M_*r*_ 83,000 and pI 8.23) was not visible on the corresponding silver-stained gel (Fig 5B), and would have required a pre-enrichment step prior to MS analysis, so its further characterization was not pursued. Despite limited protein yields of the other SLRA, we identified the two dominant alloantigens as ELSPBP1 and SAMP14, both of which were also detected as SMA in washed membranes. Although these proteins were not abundant in rafts (Fig 5B) they elicited a high immune response as indicated by their strong immunoreactivity (Fig 5A). Anti-rafts alloantisera also identified the acrosomal proacrosin binding protein sp32 (sp32) as a minor SLRA (Table 5). The disulfides reduced precursor and the processed forms of sp32 migrate on SDS-PAGE at M_*r*_ 60,000 and 32,000, respectively [54, 55], whereas in our two-dimensional gels disulfide non-reduced sp32 migrated with M_*r*_ 25,500 and a pI of 4.88 (Fig 5 and Table 6).

**Table 5:** Identification of SLRA.

Spot #	M/Z (+1	Peptide sequence	I.D. (Accession no.)	Remark
R2	966.45 1228.58	R.AVYDGOWK.Y K.YCLIEDYPR +C	Epididymal sperm binding E12 precursor (CAD62255)	Homologous to epididymal sperm binding protein 1 precursor (NP_999569)
R3	1137.58	GCVQSTXCGR	Sperm acrosome membrane-associated protein 4 precursor (NP_081331, NP_598005 and NP_001171400)	Homologous to SAMP4 of *M*. *musculus*, *H*. *sapiens*, and *S*. *scrofa*
*R4	953.60	FCTTVAVR	Lymphocyte antigen 6K (XP_020944432.1)	Peptide sequence identical to predicted tryptic peptide of Ly6K from *S*. *scrofa*
*R5	1200.27 1729.49	R.MDFWCAR.L +C K.LEQCHSETNXQR.Q +C	Proacrosin-binding protein sp32 precursor (Q29016)	

X = isoleucine (I) or leucine (L)

* de novo sequencing was performed

Peptide modifications: +C, carbamidomethyl

**Table 6:** Biochemical properties of SLRA.

Spot #	I.D.	Mr,/pI		Putative function [Reference]
		Theoretical*	Experimental**	
R2	E12 precursor/Epididymal sperm binding protein 1/ELSPBP1	26,200/7.8	23,800/6.78	Sperm-egg adhesion and capacitation [45,46]
R3	Sperm acrosome associated 4 and sperm acrosomal membrane protein 14 (SAMP4 and SAMP14)	13,004/5.49†	13,000/3.70	Egg adhesion and fusion [53]
R4	Lymphocyte antigen 6K (Ly6K)	None	18,000/5.79	Sperm transit into oviduct [54]
R5	Proacrosin-binding protein sp32 precursor	60,540/4.82	25,500/4.88	Facilitator of proacrosin conversion [55,56]

*Computed from sequence data from *Sus scrofa* where available, using Primary Structure Analysis Tools of Swiss Institute of Bioinformatics ExPASy Proteomics Server, or EditSeq (Lasergene)

**Protein disulfides not reduced

^†^Calculated using sequence data from *Homo sapiens*

One peptide from SLRA “R4” initially yielded no matches in MS/MS ion searches, and its *de novo* sequence (FCTTVAVR) yielded no matches in BLASTp comparisons to known sequences using the NCBI NR mammalian database. After this manuscript was originally submitted, new MS ion and BLASTp searches positively identified R4 as lymphocyte antigen 6k (Ly-6k; Table 5). This SLRA was initially detected as a single spot on two-dimensional immunoblot, but a longer exposure revealed a train of several immunoreactive R4 spots, suggesting that several R4 variants may exist (Fig 5, “5 min exp.” panel).

## Discussion

Our results show that alloimmunizing gilts with sperm particulate fractions enriched in plasma membranes or lipid rafts decreases their fertility, and the antigens targeted by the alloimmune response comprise fewer than 20 immunodominant proteins that include known sperm-specific proteins as well as two previously undiscovered proteins that may function in swine fertilization. Thus pig sperm membranes and lipid rafts contain a relatively small number of molecules that are potential contraceptive immunogens in swine. To our knowledge this is the first report that *in vivo* fertility of a large animal is diminished by active immunity to sperm membrane or lipid raft alloantigens. Whether this decrease in fertility is caused by antibody binding to a single or multiple target antigens remains to be determined, as both the overall intensity of individual gilts' alloimmune responses and the number of alloantigens recognized were higher in the animals with decreased fertility. Collectively, our *in vivo* fertility and targeted proteomics findings defined the repertoire of dominant membrane and lipid raft antigens that could serve, individually or in combination, as targets for rational development of an immunocontraceptive vaccine.

S1 Table summarizes the full complement of sperm-specific membrane proteins identified in this study. The major alloantigens of pig sperm membranes are fertilin α (ADAM 1), fertilin β (ADAM 2), and cyritestin (ADAM 3), sperm-specific members of the ADAM (A Disintegrin And Metalloprotease) gene family known to be important for sperm fertility. We also identified the major alloantigens of pig sperm lipid rafts (detergent-resistant membranes, DRM) as sperm acrosomal membrane protein 14 (SAMP14) and epididymal sperm binding protein E12 (ELSPBP1). The enhanced immunoreactivity of anti-rafts alloantisera with these egg binding candidates supports previous observations that they are present and potentially enriched in sperm DRM, where they may mediate cellular interaction during early events of fertilization. Our proteomics experiments also identified other sperm-specific proteins as SMA in swine, and detected two previously uncharacterized proteins, one of which was annotated only very recently in the porcine genome. Thus this study reinforces the idea that the activities of one or more SMA and SLRA are required for successful fertilization, as supported by previous protein biochemistry and gene knockout studies by others. It also shows that systematically characterizing SMA is a productive approach to identifying new proteins that may function in fertilization, as we successfully identified several putative new sperm-specific protein isoforms.

One of our most striking findings was the observation that ADAMs 1–3 are overwhelmingly the immunodominant alloantigens of pig sperm membranes. These proteins were abundant in pig sperm membrane preparations, and they have been extensively characterized in rodent spermatozoa, though their functions in fertilization are not completely understood. The founding members of the ADAM gene family were discovered in guinea pig spermatozoa [57], and for more than a decade sperm-specific ADAMs were believed to mediate sperm-egg plasma membrane fusion [58]. Subsequent studies of ADAM-deficient mice revealed that ZP adhesion and sperm migration into the oviduct are impaired instead of fusion [59–62] and that ADAMs function in protein trafficking prior to fertilization [61,62], so these proteins may not function directly in gamete interactions. Nevertheless, our results, particularly the fertility studies, support the idea that ADAM1b, ADAM2 or ADAM3 function in mammalian fertilization, at least in swine. Moreover, the mass/pI variation we observed in these proteins suggests that porcine ADAMs undergo extensive post-translational modification in their functional maturation, which may in turn reflect species differences in their structure and function.

In addition to the detection of known porcine sperm-specific proteins such as AS-A, sp47 and spermadhesins, our proteomic strategy identified three proteins as low abundance SMA's that were not previously known to alloantigenic (zymogen granule membrane glycoprotein-2, mitochondrial ATP synthase F1 β subunit, and myo-inositol monophosphatase-1). Our identification of the latter two proteins is consistent with results from previous proteomic studies of pig testis and human spermatozoa [63–68]. These three sperm proteins' alloimmunogenicity strongly suggests they are expressed in spermatozoa as structurally distinct, sperm-specific isoforms different from those found in somatic tissues (see S2 Table for further discussion of these SMA). Also, *de novo* MS/MS analysis generated peptide sequences for SMA “M6” and SLRA “R4” that initially exhibited no apparent similarity to known proteins in the NCBI NR (non-redundant) mammalian database. Subsequent analysis after the Swine Genome Sequencing Consortium deposited a large number of annotated sequences to the NCBI database that our R4 sequence was identical to a predicted tryptic peptide of porcine Ly6K (lymphocyte antigen 6K). Thus our raft antigen R4 is positively identified as Ly6K. Similarly, updated BLASTp searches identified several sequences partially matching our M6 peptide sequences. The matches are with hypothetical proteins predicted by automated annotation of genomic sequence, and include most notably an "uncharacterized proline-rich protein" from Damara mole rat (XP_010624656) and an "oleosin-B6-like" protein from rabbit (XP_008247746). Our subsequent studies of “M6” show its mRNA is indeed testis-specific and encodes a previously undiscovered, 20 kDa swine protein. The sizes of the hypothetical proteins from mole rat and rabbit are smaller than swine M6, and no tissue expression or protein biochemistry data are available for these orphan proteins, so further investigation will be needed to determine if they are indeed M6 orthologs. Moreover, detailed characterization of M6 will yield essential information about this protein's properties that will in turn provide insight into its function in fertilization.

Lipid rafts are microdomains of the plasma membrane that mediate many cell-cell interaction processes because they are rich in glycosylphosphatidylinositol (GPI)-anchored proteins and various signal transduction complexes [24–26]. Physical methods confirmed presence of nanoscale planar domains in the cell membrane of living *B*. *subtilis* [69], consistent with existence of lipid rafts *in vivo*. Numerous studies implicate lipid rafts as platforms for cell adhesion and signaling molecules [25,26,70,71], and lipid rafts are present in sperm membranes of various species from sea urchin to swine and human [27–31,72] where they may contribute to the localization, organization, and regulation of specific signaling pathways. Consequently, DRM may be an enriched source of rare sperm surface proteins with possible functions in fertilization. Our identification of an M_*r*_ 25,500 proacrosin-binding protein sp32 in DRM suggests this soluble, acrosomal protein [55,56] also exists as a functionally unique, membrane-associated variant, consistent with the prior detection of sp32 isoforms in lipid rafts isolated from boar spermatozoa [72]. Furthermore, the presence of several egg-binding candidates in DRM [31,66,72,73] supports the idea that sperm lipid rafts and raft proteins are functionally important in fertilization, particularly zona pellucida (ZP) recognition and adhesion. Indeed, the mouse DRM protein GLIPR1 (glioma pathogenesis-related 1) redistributes to the anterior sperm head during capacitation, and antibodies to GLIPR1 inhibit sperm-ZP interactions [74]. Our identification of porcine epididymal sperm protein E12 (ELSPBP1) as a major alloantigen of DRM, as revealed by the strong immunoreactivity of its low abundance charge isoforms, is likewise consistent with a possible function for this fibronectin type-II (Fn2)-module protein in sperm-ZP adhesion [44,45]. In addition, identification of the porcine SAMP14 ortholog as a major SLRA supports previous results suggesting that human SAMP14 functions as an egg recognition protein during sperm-egg adhesion [51]. Because SAMP14 and ELSPBP1 may function in sperm-egg interaction, these DRM proteins, as well as the other membrane alloantigens we identified, are potential targets for immunocontraceptive vaccines, especially considering we observed high ELSPBP1 immunoreactivity in animals that produced smaller litters. Finally, our identification of raft antigen R4 as Ly6K further validates our rafts preparation, as Ly6K is a GPI-anchored protein expressed predominantly in male germ cells [75, 76] that associates with TEX101 [77], itself too a GPI-anchored glycoprotein expressed in male (as well as female) germ cells. Genetic loss of Ly6K in mice impairs sperm transit into the oviduct, resulting in male infertility [54]. Interestingly, Ly6K is a 'cancer-testis antigen' expressed at elevated levels in various cancers [78], and a promising target for anticancer vaccine production [79], thus establishing its general suitability as a vaccine antigen that might also prove useful in an immunocontraceptive formulation.

Though this study identified many immunodominant antigens that are potentially suitable targets for immunocontraception, some questions remain unanswered. We did not identify low abundance or weakly immunogenic sperm-specific proteins that could nonetheless be good targets. Also, it is unclear why sperm membranes induced strong alloimmune responses in only two of four immunized animals. Because of their size and strength, gilts are more difficult to inject than small lab animals, so a portion of the antigen depot may simply have been lost in the low responders. Alternatively, the amount of antigen injected may not have been sufficient to induce uniformly strong and persistent immune responses. Genetic differences affect immune response to sperm and testis antigens [10–12], so some response variation is expected among outbred animals, especially when immunogen is limited. A limiting immunogen dosage could also explain the incomplete loss of fertility (73%) in the high responder animals. We immunized gilts weighing ~100 kg with 80 mg of sperm membrane protein, which scales to immunization of a mouse with only 16 μg of a complex protein mixture. Considering the comparatively small amount of injected antigen preparation relative to the sizes of the gilts, the achieved decrease in fertility seems remarkable. And though partial loss of fertility would not be acceptable for human immunocontraception, it could be effective for control of pest species including feral swine. Indeed, a vaccine that produces subfertility might be ideal for controlling populations of species such as elephants, horses, or bison that are desirable in moderate numbers but destructive when too numerous [80]. Injecting a larger quantity of immunogen would likely induce stronger responses and greater loss of fertility, but doing so is not feasible with whole membranes or DRM because of the amounts of spermatozoa needed. Regardless, vaccine refinement and optimization can readily be accomplished using synthetic fragments or expressed recombinant forms of the identified target antigens [81], either individually or in combinations, to assure induction of uniformly strong immune responses that in turn produce effective and sustained contraceptive effects in outbred populations.

In summary, this is the first study to use a targeted proteomics approach to identify sperm membrane and lipid rafts alloantigens and thereby systematically define the repertoire of major sperm-specific membrane proteins in a single species. It is also the first to show: 1) ADAM proteins overwhelmingly dominate the alloimmune response to pig sperm membranes; 2) three known proteins from somatic cells (ATP synthase F1 ß subunit, myo-inositol monophosphatase-1 and zymogen granule membrane glycoprotein-2) are expressed as alloantigenic, sperm-specific isoforms; 3) sperm membranes contain two previously undiscovered sperm-specific proteins (SMA “M6” and SLRA “R4”) that may have unique functions in fertilization; and 4) alloimmunity to sperm membranes or lipid rafts significantly decreased the fertility of gilts, suggesting that at least one of the identified SMA or SLRA, individually or in combination with others, functions in fertilization. Ultimately, these findings improve our understanding of gamete interactions by defining the repertoire of sperm-specific proteins that mediate key cellular events during fertilization, and identify targets for development of molecular methods for evaluating and regulating fertility in various animal species including humans.

## Supporting information

S1 Fig Flow chart illustrating the two procedures used for isolation of pig sperm particulate fractions containing released plasma membranes. Method 1 was modified from Haden et al. (2000) and Bi et al. (2003), and Method 2 from Buhr et al. (1989) and Flesch et al. (1998).
(DOC)

S2 Fig Flow chart illustrating the procedure used for immunization of gilts, production of new alloantisera to sperm membranes (anti-TWM #2, pool of high responder gilts 466 and 474) and lipid rafts, and fertility trial. (DOC)

S3 Fig Purity of the sperm particulate fraction containing released plasma membranes assessed by western blot analysis. Immunoblots (representative of at least 3 replicate experiments) showing efficiency of high-salt washes (1M) at removing the **A)** acrosomal matrix protein proacrosin from membrane preparations isolated with two different methods, and **B)** acrosomal protein zonadhesin from membrane preparations isolated by Method 1 or 2. Equal volumes of supernatant solution after ultracentrifugation (W1-W3, washes #1–3) were precipitated with acetone at -20°C prior to SDS-PAGE. Twenty μg of triple-washed membrane (TWM) proteins were solubilized in non-reduced sample buffer (10 mM Tris-HCl pH 6.8, 2% SDS, 15% glycerol), heated at 65°C for 5 minutes, separated by SDS-PAGE (12% or 8–15% linear gradient), and electrotransferred to nitrocellulose or PVDF membrane in transfer buffer (250 mM Tris-HCl pH 7.5, 192 mM glycine, ±0.001% SDS) containing 10–20% methanol (50 V; 1.5 h; 4 or 23°C). Nonspecific binding sites were blocked in 2% milk powder-TBST (10 mM Tris-HCl pH 7.5, 150 mM NaCl, 0.1% Tween 20) and the blots incubated at 23°C with anti-proacrosin (1/40,000) or anti-zonadhesin (1/20,000) in TBST. Blots were then washed (3 times, 10 min per wash) in TBST, incubated 45 min with horseradish peroxidase (HRP)-conjugated protein A diluted 1/10,000 in milk-TBST, or HRP-goat anti-rabbit antibody diluted 1/50,000 in TBST, washed with TBST as above, and immunoreactive proteins detected as described in the “Materials and Methods”.
(DOC)

S4 Fig Distribution of lipid rafts and their protein and ganglioside G_M1_ contents in the sucrose density gradient of sperm Triton X-100 extract. Lipid rafts in detergent extracts of boar spermatozoa were isolated by sucrose gradient ultracentrifugation as described in “Materials and methods”. Numbers in the *x* axis represent 1-ml fractions from the top (#1) to the bottom (#12) of the tube. Raft fractions (#4 and 5) are *boxed* in dashed lines. **A)** Light scattering at 620 nm of each fraction (top panel), and total amount of proteins in μg in each fraction (bottom panel). Data are expressed as mean± SD from 6 different lipid raft preparations with semen from 4 different boars. **B)** Dot blot representative of 6 replicate experiments showing the profile of G_M1_ within the sucrose density gradient by dot blot immunoassay (modified from Nixon et al., 2009). 100 μl of each fraction (n = 4 lipid raft preparations) were added to wells of a Dot Blot apparatus (Schleicher & Schuell, Keene, NH), and suctioned onto nitrocellulose membranes. After washing each well with PBS, the membranes were air dried, blocked for 1 hour with 5% w/v milk powder in TBS containing 0.1% Tween 20 (TBST), then incubated for 1 hour with HRP-conjugated cholera toxin beta subunit (CTB) diluted at 0.06 μg/ml in TBST containing 0.5% w/v milk powder. After three washes (10 min each) with TBST, dot blots were developed as described in the “Materials and Methods”. **C)** Immunoblot representative of 4 replicate experiments showing distribution of the raft marker flotillin-2 within the sucrose density gradient by immunoblotting. A volume of fraction 4 containing 40 μg of proteins was precipitated with MeOH/CHCl_3_ (Wessel and Flugge, 1984). For fractions 1–3 and 5–9, the same volume as fraction 4 was precipitated, and for fractions 10–12, 1/5 of the volume of fraction 4 was precipitated. Lane B, 5 μg protein form mouse brain as positive control for flotillin-2. Raft proteins were solubilized and resolved by SDS-PAGE (12% gel, disulfides not reduced), then transferred to PVDF membrane. Blots were blocked 1h in TBST and 30 min in 2% milk-TBST, incubated overnight (23°C) with anti-flotillin-2 (1/1000 in TBST). After washing with TBST, bound flotillin antibody was detected with HRP-conjugated anti-mouse IgG (1/2000 in TBST, 1 h, 23°C). Antigen-antibody complexes were detected as described in “Material and methods”.
(DOC)

S1 Table Summary of SMA and SLRA identified by MS throughout the proteomic study. (DOC)

S2 Table Summary of identified SMAs’ properties. (DOC)
