# Supplementary material for: Immunocontraceptive target repertoire defined by systematic identification of sperm membrane alloantigens in a single species
Source: PLoS One. 2018 Jan 17;13(1):e0190891. doi: 10.1371/journal.pone.0190891 (PMC5771590; doi:10.1371/journal.pone.0190891)
Supplement: S2 Table — (DOC) [file pone.0190891.s006.doc]

**S2 Table**

| **ATP synthase F1 ß subunit**  Proteomic studies identified the mitochondrial ATP synthase ß chain in testis [1] and spermatozoa [2-5]. Whether this protein is present in plasma membrane of somatic cells is controversial [6,7], though it has been identified in DRMs of mouse spermatozoa [3], and reported to have extra-mitochondrial localization in the flagellar midpiece and equatorial segment of hamster spermatozoa [8,9]. These studies suggest ATP synthase F1 ß subunit may function in early events of fertilization such as motility and acrosome reaction. It is possible that the membrane-enriched particulate fraction we used for proteomic analysis contained parts of the sperm tail including the midpiece with its associated mitochondria. |
| --- |
| ***myo*-Inositol monophosphatase**  Inositol phosphate signaling occurs in the acrosome reaction (AR), so the sperm-specific *myo*-inositol monophosphatase may function in the regulation of this exocytotic event. Mice deficient for a newly identified muscle-specific inositol phosphatase (MIP/ MTMR14) exhibited diminished dephosphorylation activity, and subsequent accumulation of MIP/MTMR14 substrates (PtdInsP_2_) in MIP^-/-^ mice resulted in spontaneaous Ca^2+^ leakage from the internal store-sarcoplasmic reticulum [10]. In the the AR, *myo*-inositol monophosphatase may dephosphorylate precursors upstream of PtdIns(1,4,5)P_3_, such as PtdInsP and PtdIns(4,5)P_2_, and thereby prevent precocious AR in the absence of a physiological inducer. |
| **Zymogen granule membrane glycoprotein-2 (GP-2)**  GP-2, a major component of the zymogen granule membrane of the exocrine pancreas with sequence similarity to uromodulin/ Tamm-Horsfall protein [11], is GPI-anchored to membranes. It contains a ZP domain, which is a conserved module for polymerization of extracellular proteins [12]. Accordingly, pancreatic GP-2 forms high molecular mass aggregates, so the *M*r 125,000 polypeptide we detected on two-dimensional western blot may represent a homodimer. These features further suggest GP-2 may interact with proteins containing von Willebrand D-domains, such as zonadhesin, and thus have a unique function in egg interaction and/or adhesion during early events of fertilization. A microarray study reported the expression of the GP-2 mRNA in the boar epididymis, and described a partial cDNA sequence of GP-2 encoding an amino acid sequence similar to our GP-2 sequence obtained by *de novo* sequencing [13]. |
| **Arylsulfatase A, AS-A (or SLIP1 in mouse)**  Our two-dimensional analysis detected four isoforms of AS-A not previously described in boar spermatozoa. Post-translational modifications such as glycosylation may explain the p*I* variants, whereas electrophoretic mobility variants may reflect differences in electrophoresis conditions (e.g. disulfides reduced vs. nonreduced), as disulfide-reduced proteins typically migrate at a slower rate in SDS-PAGE and thus yield a higher calculated M_r_ than non-reduced proteins. |
| **Sperm acrosome membrane-associated protein 1, SAMP1 (or SAMP32 in human);**  **Epididymal sperm binding protein E12, ELSPBP1**  The M_r_ of porcine SAMP1 and ELSPBP1 differed from that reported for the human protein. Electrophoresis differences, particularly disulfide bond status, as well as species differences of amino acid sequences may account for the apparent M_r_ discrepancies between porcine and human orthologs [14,15]. |
| **Sp47, DQH, and spermadhesins AWN-1 and AQN-3**  These peripheral proteins do not have a testicular origin, yet high (1M) salt washes did not remove them, so they were very strongly associated with the sperm plasma membrane. It might have been beneficial to wash our membrane preparations with a combination of high salt and high pH (≥11), which can be more effective than high salt alone at removing non-membrane and membrane-associated proteins [16,17], considering our primary goal was to discover integral membrane proteins most likely to be receptor/ adhesion/signaling molecules. Nevertheless, these proteins' strong association with sperm membranes suggests they could be present on the sperm surface at the time and site of fertilization *in vivo*. |

**S2 Table references**

1. Huang SY, Lin JH, Chen YH, Chuang CK, Lin EC, Huang MC, et al. A reference map and identification of porcine testis proteins using 2-DE and MS. Proteomics 2005;5: 4205-4212.

2. Martinez-Heredia J, Estanyol JM, Ballesca JL, Oliva R. Proteomic identification of human sperm proteins. Proteomics 2006;6: 4356-4369.

3. Nixon B, Bielanowicz A, McLaughlin EA, Tanphaichitr N, Ensslin MA, Aitken RJ. Composition and significance of detergent resistant membranes in mouse spermatozoa. J Cell Physiol. 2009;218: 122-134.

4. Peddinti D, Nanduri B, Kaya A., Feugang JM, Burgess SC, Memili E. Comprehensive proteomic analysis of bovine spermatozoa of varying fertility rates and identification of biomarkers associated with fertility. BMC Syst Biol 2008;2: 19.

5. Zhao C, Guo XJ, Shi ZH, Wang FQ, Huang XY, Huo R, et al. Role of translation by mitochondrial-type ribosomes during sperm capacitation: an analysis based on a proteomic approach. Proteomics 2009;9: 1385-1399.

6. Kim T, Oh J, Woo JM, Choi E, Im SH, Yoo YJ, et al. Expression and relationship of male reproductive ADAMs in mouse. Biol Reprod 2006;74: 744-750.

7. Bae TJ, Kim MS, Kim JW, Kim BW, Choo HJ, Lee JW, et al. Lipid raft proteome reveals ATP synthase complex in the cell surface. Proteomics 2004;4: 3536-3548.

8. Fujinoki M, Kawamura T, Toda T, Ohtake H, Ishimoda-Takagi T, Shimizu N, et al. Identification of 36-kDa flagellar phosphoproteins associated with hamster sperm motility. J Biochem 2003;133: 361-369.

9. Fujinoki M, Kawamura T, Toda T, Ohtake H, Shimizu N, Yamaoka S, Okuno M. Identification of the 58-kDa phosphoprotein associated with motility initiation of hamster spermatozoa. J Biochem 2003;134: 559-565.

10. Shen J, Yu WM, Brotto M, Scherman JA, Guo C, Stoddard C, et al. Deficiency of MIP/MTMR14 phosphatase induces a muscle disorder by disrupting Ca(2+) homeostasis. Nat Cell Biol 2009;11: 769-776.

11. Fukuoka S, Freedman SD, Yu H, Sukhatme VP, Scheele GA. GP-2/THP gene family encodes self-binding glycosylphosphatidylinositol-anchored proteins in apical secretory compartments of pancreas and kidney. Proc Natl Acad Sci U S A 1992;89: 1189-1193.

12. Jovine L, Darie CC, Litscher ES, Wassarman PM. Zona pellucida domain proteins. Annu Rev Biochem 2005;74: 83-114.

13. Guyonnet B, Marot G, Dacheux JL, Mercat MJ, Schwob S, Jaffrezic F, Gatti JL. The adult boar testicular and epididymal transcriptomes. BMC Genomics 2009;10: 369.

14. Hao Z, Wolkowicz MJ, Shetty J, Klotz K, Bolling L, Sen B, et al. SAMP32, a testis-specific, isoantigenic sperm acrosomal membrane-associated protein. Biol Reprod 2002;66: 735-744.

15. Ekhlasi-Hundrieser M, Schafer B, Philipp U, Kuiper H, Leeb T, Mehta M, et al. Sperm-binding fibronectin type II-module proteins are genetically linked and functionally related. Gene 2007;392: 253-265.

16. Speers AE, Wu CC. Proteomics of integral membrane proteins-theory and application. Chem Rev 2007;107: 3687-3714.

17. Josic D, Clifton JG. Mammalian plasma membrane proteomics. Proteomics 2007;7: 3010-3029.
